# Corticosterone Enhances the AMPK-Mediated Immunosuppressive Phenotype of Testicular Macrophages During Uropathogenic *Escherichia coli* Induced Orchitis

**DOI:** 10.3389/fimmu.2020.583276

**Published:** 2020-12-08

**Authors:** Zhengguo Zhang, Ziming Jiang, Yiming Zhang, Yu Zhang, Yan Yan, Sudhanshu Bhushan, Andreas Meinhardt, Zhihai Qin, Ming Wang

**Affiliations:** ^1^ Department of Urology, The First Affiliated Hospital of Zhengzhou University, Zhengzhou, China; ^2^ Medical Research Center, The First Affiliated Hospital of Zhengzhou University, Zhengzhou, China; ^3^ Department of Anatomy and Cell Biology, Justus-Liebig-University of Giessen, Giessen, Germany; ^4^ School of Basic Medical Sciences, The Academy of Medical Sciences of Zhengzhou University, Zhengzhou, China

**Keywords:** Corticosterone, Testicular macrophage, AMPK, Fatty acid oxidation (FAO), Orchitis

## Abstract

Testicular macrophages (TM) play a central role in maintaining testicular immune privilege and protecting spermatogenesis. Recent studies showed that their immunosuppressive properties are maintained by corticosterone in the testicular interstitial fluid, but the underlying molecular mechanisms are unknown. In this study, we treated mouse bone marrow-derived macrophages (BMDM) with corticosterone (50 ng/ml) and uncovered AMP-activated protein kinase (AMPK) activation as a critical event in M2 polarization at the phenotypic, metabolic, and cytokine production level. Primary TM exhibited remarkably similar metabolic and phenotypic features to corticosterone-treated BMDM, which were partially reversed by AMPK-inhibition. In a murine model of uropathogenic *E. coli-*elicited orchitis, intraperitoneal injection with corticosterone (0.1mg/day) increased the percentage of M2 TM *in vivo*, in a partially AMPK-dependent manner. This study integrates the influence of corticosterone on M2 macrophage metabolic pathways, phenotype, and function, and highlights a promising new avenue for the development of innovative therapeutics for orchitis patients.

## Introduction

Urogenital infection and immunological factors account for 13 to 15% of all cases of male infertility (1, 2). These are potentially curable or treatable conditions, but as yet sufficient knowledge of the underlying pathology or possible therapeutics is scarce. The testis is an immune-privileged organ that needs to tolerate autoantigens of germ cells and maintain immune homeostasis even under infectious or inflammatory conditions (3, 4). Testicular macrophages (TM), the main population of immune cells in the testis, play a key role in the maintenance of immune privilege (5–7), however, how they balance this function with defense against invading pathogens is largely unknown.

Much of our knowledge on TM has come from rat models, where these cells are largely anti-inflammatory, exhibiting reduced pro-inflammatory responses and an immunosuppressive phenotype, while retaining their phagocytic capacity (8, 9). Rat TM maintain their immunosuppressive function by secreting high amounts of the anti-inflammatory cytokine IL-10, and only low levels of pro-inflammatory cytokines such as TNF-α and IL-6 (10, 11). Most recently, corticosterone in the testicular interstitial fluid (TIF) was identified as an important mediator maintaining TM function and phenotype (12).

Clinically, corticosterone is a widely used immunosuppressive drug that belongs to the glucocorticoid family of steroid hormones, and elicits a wide range of biological effects including immunosuppressive and anti-inflammatory actions (13–15). *In vivo* corticosterone is mainly synthesized in the adrenal glands, and enters target organs *via* the bloodstream. However, corticosteroids can also be produced in extra-adrenal tissues such as the skin and intestine (16). In this regard, we found that corticosterone levels in TIF were significantly higher than in serum, even in rats following adrenalectomy (12). We uncovered significant intratesticular production of corticosterone by TM, suggesting an autocrine loop of corticosterone action in the testis. However, the underlying mechanisms of how corticosterone maintains the immunosuppressive function of TM and what the effects of corticosterone are on TM in infectious conditions are still not fully understood.

Two broad types of macrophages exist in tissues: classically activated pro-inflammatory M1 macrophages, and alternatively activated anti-inflammatory M2 macrophages. However, these phenotypes are not fixed, and most recently studies have revealed the importance of cellular metabolism in fate decision of macrophages. M1 and M2 macrophages rely on different metabolic pathways, namely glycolysis and oxidative phosphorylation, respectively, to maintain their specific phenotypes and functions (17, 18). Accordingly, a switch between these metabolic pathways can induce the alternation of macrophage phenotype: inhibiting oxidative phosphorylation not only suppresses the M2 macrophage state, but actively induces M1 macrophage polarization (19); while in contrast, activation of oxidative phosphorylation and PGC-1β reduces pro-inflammatory cytokine production in M1 macrophages (20). AMP-activated protein kinase (AMPK), a master-regulator of cellular and systemic energy homeostasis, has been identified as the key mediator of inflammatory signaling pathways in macrophages (21). AMPK activation facilitates macrophage differentiation to the anti-inflammatory M2 phenotype (22). Given the finding that corticosterone could impair the oxidative energy metabolism of the liver mitochondria (23), and based on these recent studies, we speculate that corticosterone could mediate the TM phenotype by altering their metabolic pathways.

Here we aimed to bring together recent findings on rat TM, corticosterone, and macrophage metabolism to ask about the pathways driving and maintaining the anti-inflammatory polarization of murine TM. Studying bone marrow derived monocytes (BMDM) and primary TM *in vitro* we found that corticosterone induced the activation of AMPK and fatty-acid oxidation pathways in BMDM, leading to M2 polarization, which phenocopied the anti-inflammatory features of TM. Similarly, *in vivo* in a uropathogenic *Escherichia coli* (UPEC)-elicited orchitis model, corticosterone treatment prevented UPEC-induced decreases in M2 TM in the mouse testis, which was partly AMPK-dependent.

## Materials and Methods

### Animals

All mice were purchased from Charles River Laboratories (China). Animal experiments were conducted in accordance with the recommendations specified in the guide for the care and the protocols approved by the Ethics Committee of Zhengzhou University (Ethics Number: KY154). Mice (C57BL/6J, 8–10 weeks, male) were housed in groups of five in individually ventilated cages under a cycle of 12 h of light and 12 h of dark, in specific pathogen-free conditions with constant temperature (21°C) and 50–60% humidity. Mice were killed by isoflurane, and tissues were rapidly collected.

### UPEC Orchitis Model

The bacteria-induced orchitis model was established as described previously (24). Briefly, after general anesthesia, a scrotal incision was made to expose the testes, epididymidis, and vasa deferentia. Ten µl of UPEC strain 536-saline (0.9% sodium chloride solution) suspension (about 5 × 10^5^ bacteria) were injected bilaterally into the vas deferens proximal to the cauda epididymis using 30 G needles. Sham operated mice were injected with saline. The vasa deferentia were ligated close to the site of injection to prevent spreading of infection anterograde towards the urethra. From the second day after the operation, animals were intraperitoneally injected with corticosterone (0.1mg/day) and or Compound C (0.4 mg/kg, ABSIN, China, Shanghai). Mice were kept under standard housing conditions until being sacrificed at day 7 post operation. Both testes were removed aseptically for further analysis by flow cytometry or H&E staining.

### Primary TM and PM Isolation

TM and PM were isolated from adult C57BL/6J mice as previously described (25, 26). Briefly, testes were decapsulated and collected into 10 ml of ice-cold endotoxin-free RPMI 1640 medium. The seminiferous tubules were gently separated as Hayes et al. described (27). The volume was adjusted to 50 ml and fragments were allowed to settle for 5 min, then the supernatant was recovered and centrifuged at 300 g for 10 min at 4°C. The interstitial cell pellet was resuspended in 5 ml RPMI 1640 culture medium and adjusted to a concentration of 5 × 10^6^ cells/ml. After several washes with PBS, cells (2 × 10^6^/well) were plated into six-well plates and incubated at 37°C in an atmosphere containing 5% CO_2_ for 30 min. Contaminating cells were removed by extensive washing. Peritoneal exudate cells were harvested by lavage with cold RPMI 1640 medium (10 ml per mouse). Cells were sedimented at 300 g for 10 min at room temperature. The cell pellet was resuspended in 10 ml medium, and 2 × 10^6^ cells/well were seeded into six-well plates and incubated at 37°C in an atmosphere containing 5% CO_2_ for 30 min to allow PM to adhere. Non-adherent cells were removed by washing thoroughly three times with RPMI 1640. Purity of TM and PM were analyzed by flow cytometry using antibodies against the macrophage markers F4/80 and CD11b, and found to be approximately 75–80% (TM) and 90% (PM), respectively (**Supplementary Figure 1**).

### BMDM Isolation and Culture

Bone marrow-derived macrophages (BMDM) were generated from the femur and tibia of adult male mice. The bone marrow was rinsed into phosphate buffer saline (PBS) with a 1 ml syringe and centrifuged at 300 g for 5 min at RT. The cell pellet was resuspended in 5 ml red blood cells lysis buffer for 3 min to lyse the erythrocytes, then centrifuged at 300 g for 5 min at RT before being resuspended again in RPMI-1640 medium supplemented with 10% FBS and 1% penicillin/streptomycin. The cell concentration was adjusted to 5 × 10^6^ cells/ml; 2 × 10^6^ cells/well were seeded onto 12-well plates and incubated at 37°C in an atmosphere containing 5% CO_2_ for 30 min. Contaminating cells were removed by extensive washing, taking advantage of the rapid adherence of macrophages. Finally, the isolated cells were cultured with RPMI 1640 medium containing 10% FBS, 1% penicillin/streptomycin, and 50 ng/ml granulocyte-macrophage colony-stimulating factor (GM-CSF, Biolegend, San Diego, USA).

### ELISA

The ELISA Ready-SET-GO-Assays (TNFα, Cat#: 430901, Biolegend; IL-10, Cat#: 431411, Biolegend) were used to measure the concentrations of TNFα and IL-10 protein in BMDM and primary macrophage culture medium, according to the manufacturer’s instructions.

### RT-PCR

Total RNA was extracted from cells using Trizol Reagent. Complementary DNA was synthesized from 2 μg total RNA using for cDNA reverse transcription. Prime Script RT Master Mix (Applied TaKaRa, Otsu, Shiga, Japan) was used in accordance with the manufacturer’s instructions. qRT-PCR was performed on an ABI PRISM 7300HT Sequence Detection System (Applied Biosystems, Foster City, CA, USA) using SYBR Green PCR Master Mix (TaKaRa). The primers used are listed in **Table 1**. The average threshold cycle number (CRtR) for each tested mRNA was used to quantify the relative expression according to the 2^−ΔΔ^ *^C^* ^t^ method, and *Gapdh* was used as an internal control.

**Table 1:** The primers used in this paper.

Genes	Forward	Reverse
*Tnfa*	AAAGACCAGGTGGAGTGGAAGAAC	CTCAGTGCCGATGGAGTCCGAGTA
*Il10*	CCCATTCCTCGTCACGATCTC	TCAGACTGGTTTGGGATAGGTTT
*Acc1*	ACAGTGGAGCTAGAATTGGAC	ACTTCCCGACCAAGGACTTTG
*Cpt1*	CAGAGGATGGACACTGTAAAGG	CGGCACTTCTTGATCAAGCC
*Acadm*	TGGCATATGGGTGTACAGGG	CCAAATACTTCTTCTTCTGTTGATCA
*Cd36*	ATTGGTCAAGCCAGCT	TGTAGGCTCATCCACTAC
*Fasn*	AGCGGCCATTTCCATTGCCC	CCATGCCCAGAGGGTGGTTG
*Acsl1*	TCCTACAAAGAGGTGGCAGAACT	GGCTTGAACCCCTTCTGGAT
*Gapdh*	TCTCTGCTCCTCCCTGTTCC	TACGGCCAAATCCGTTCACA

### Western Blotting

Cell lysates were prepared in RIPA buffer (65 mM Tris-HCl, 150 mM NaCl, 1 mM EDTA, 1% NP-40, 0.5% sodium deoxycholate, 0.1% SDS, and protease inhibitor cocktail) and protein concentrations were measured by the Bio-Rad Protein Assay (Bio-Rad, Hercules, USA). Twenty μg of protein from each sample were subjected to SDS-PAGE and transferred onto nitrocellulose membranes. The membranes were incubated with 5% non-fat milk in TBST (0.5% Tween 20) for 1 h and washed three times with TBST washing buffer. After incubation with primary antibodies against p-AMPK (2535s, CST, Danvers, USA), AMPK (5831s, CST), p-ACC1 (3661s, CST), ACADM (118183, Abcam, Cambridge, UK), or ACADVL (118183, Abcam) at 4°C overnight, the membranes were washed and then incubated for 1h with HRP-conjugated anti-rabbit or anti-mouse secondary antibodies at room temperature. The blots were washed three times with TBST and developed with an ECL system (Bio-Rad). Actin (CST) was used as a loading control.

### Flow Cytometry

To measure macrophage marker levels in primary TM and corticosterone-polarized BMDM flow cytometry was used. In brief, 1 × 10^6^ cells were washed twice with washing buffer (1% BSA in PBS) and incubated with an anti-mouse CD16/32 antibody (553142, BD Biosciences, San Jose, USA) for 10 min. Then, anti-mouse CD45 (103128, Biolegend, APC-R700, 1:100), F4/80 (123118, Biolegend, APC-cy7, 1:100), CD11b (553310, BD, FITC, 1:100), and CD206 (141716, Biolegend, Percp 5.5, 1:100) antibodies were used to identify the macrophage populations. After incubating the cells with CD45, CD11b, F4/80 for 30 min on ice, cells were washed with PBS 1% w/v BSA, fixed, permeabilized, and labeled with anti-CD206 at 4°C for 30 minutes. Then cells were washed and re-suspended in washing buffer. Flow cytometric analysis was performed by using a FACS AriaII (BD) and data were analyzed with FlowJo software version X (Tree Star, Ashland, OR, USA).

### FAO Enzyme Measurements

Analysis of fatty acid oxidation (FAO) was performed using a FAO flow cytometry kit (118183, Abcam). Levels of the FAO cycle enzymes ACADVL and ACADM were determined according to the manufacturer’s instructions.

### Seahorse XF96 Analysis

The Seahorse XF96 Extracellular Flux analyzer (Agilent, Santa Clara, USA) is a sensitive, high-throughput instrument that takes real-time measurements of respiration rates of cells with or without oxidative stress. Briefly, TM, PM, or corticosterone treated BMDM (10^4^ cells/well) were seeded on Seahorse culture plates in assay medium (DMEM, 1% BSA, 25 mM glucose, and 2 mM glutamine, 1 mM pyruvate) and analyzed using a Seahorse XFe-96 system. For measuring extracellular acidification rate (ECAR), macrophages were stimulated accordingly with 10 mM glucose, 0.25 μM oligomycin (Calbiochem, Merck, Darmstadt, Germany), and 2-deoxyglucose (2-DG, 100 mM). To obtain the maximal respiratory and control values of cells, macrophages were stimulated with oligomycin (0.25 μM), Carbonyl cyanide 4-(trifluoromethoxy) phenylhydrazone (FCCP, 1 μM), and rotenone (1 μM)/antimycin A (1 μM) according to a standard protocol (28). The cell numbers were normalized to the cell protein concentrations. ECAR and OCR were measured with the XF96 Extracellular Flux Analyzer.

### Histological Analysis

UPEC induced orchitis testes were collected and fixed in Bouin’s fixative for 4 h and then embedded in paraffin. Tissue sections (6 µm) were stained with hematoxylin and eosin. After staining, images were acquired with a Vectra microscope (Akoya Biosciences, Menlo Park, USA).

### Statistical Analyses

Statistical significance was calculated by Welch’s t-test when comparing two groups or by one-way ANOVA. A p-value <0.05 was considered statistically significant.

## Results

### Corticosterone Induces an Anti-Inflammatory Macrophage Phenotype *In Vitro*

**Figure 1:**
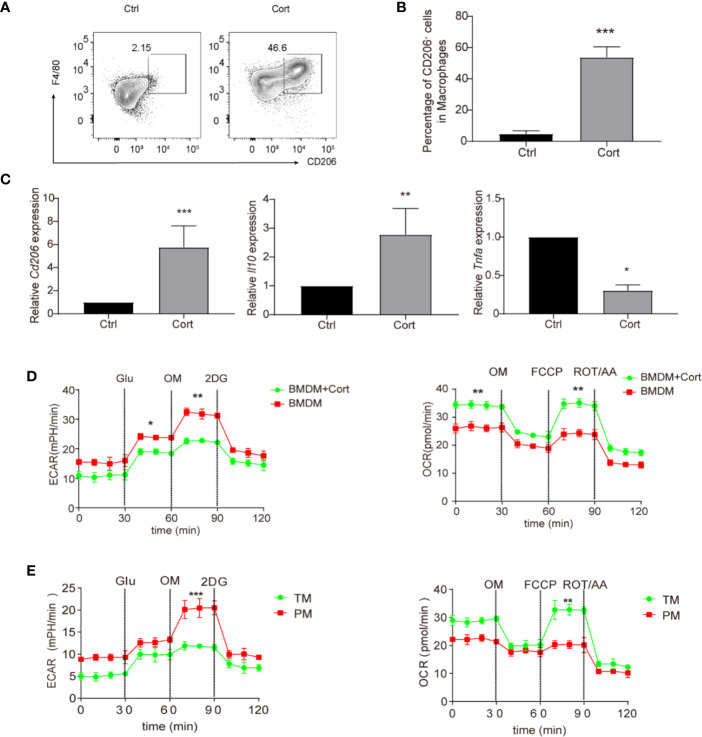
Corticosterone induces M2 macrophage polarization in BMDM. **(A–D)** BMDM were derived by culture in RPMI 1640 medium supplemented with GM-CSF (50 ng/ml), before the addition of corticosterone (50 ng/ml) for 7 days. **(A, B)** The macrophage markers F4/80 and CD206 were identified by flow cytometry. Representative plots are shown **(A)**; alongside average percentage of macrophages expressing CD206 **(B)**. **(C)** The mRNA expression levels of *Cd206, Il10*, and *Tnfa* were quantified by qRT-PCR and normalized against *Actin*. **(D, E)** BMDM cultured with or without corticosterone **(D)**, and primary TM and PM **(E)** were seeded on XF96- Seahorse culture plates (10^4^ per well). The ECAR and OCR were tested in XF-96 assay medium (see *Methods*) and normalized against protein concentration. After establishing a baseline, glucose (Glu, 10 mM), oligomycin (OM, 0.25 μM), 2-deoxyglucose (2-DG, 100 mM), or oligomycin (OM, 0.25 μM), FCCP (1 μM) and rotenone/antimycin A (ROT/AA, 1 μM) were sequentially added. Continuous ECAR and OCR values (pmoles/min/µg protein) are shown. Each repetition involved 4–5 mice per group and three replicates were performed (mean ± SD, Welch’s t-test). *P < 0.05, **P < 0.01, ***P < 0.001.

Our previous study showed that corticosterone polarized GM-CSF-induced rat blood monocytes towards the anti-inflammatory M2 macrophage phenotype (12). To understand whether parallel effects occurred in murine macrophages, we first cultured mouse BMDM with 50 ng/ml of corticosterone for 7 days. As in rats, corticosterone treatment induced high levels of expression of the M2-marker CD206 on around half of macrophages (**Figures 1A, B**). Similarly, the relative mRNA level of *Cd206* was significantly higher in corticosterone-treated cultures, as was expression of the anti-inflammatory cytokine *Il10*; whereas expression of the pro-inflammatory cytokine *Tnfa* was significantly decreased by corticosterone treatment (**Figure 1C**). As macrophage polarization and pro-/anti-inflammatory cytokine production have recently been linked to changes in cellular metabolism, we next asked whether corticosterone was altering the metabolic pathways in murine BMDM. We cultured BMDM with or without corticosterone and assessed activity of the M1-linked glycolytic pathway and M2-linked oxidative phosphorylation (OXPHOS) by measuring the extracellular acidification rate (ECAR) and oxygen consumption rate (OCR), respectively (**Figure 1D**). Corticosterone significantly increased OCR and reduced ECAR in BMDM compared with the non-treated group (**Figure 1D**), which is consistent with the increased expression of M2 phenotypic markers and cytokines induced by corticosterone treatment seen above. Thus, corticosterone induces M2 polarization at the phenotypic, molecular, and metabolic level in murine BMDM *in vitro*.

We then asked how the effects of corticosterone on BMDM metabolism compared to the metabolic profiles of macrophages in the murine testis. We isolated an enriched population of primary TM (75–80% purity) and compared their ECAR/OCR profiles to those of primary PM (approximately 90% purity), which are known to have an M1 phenotype, and to the data from BMDM (**Figure 1D**). We found that the enriched TM population exhibited increased OCR and reduced ECAR relative to PM (**Figure 1E**), and that their metabolic profile closely resembled that of corticosterone-treated BMDM (**Figure 1D**). Taken together, an enriched primary TM population and corticosterone-treated BMDM both show comparably higher levels of OXPHOS and lower levels of glycolysis, consistent with an M2 anti-inflammatory phenotype.

### Corticosterone Activates AMPK and Reprograms Fatty Acid Metabolism in BMDM

**Figure 2:**
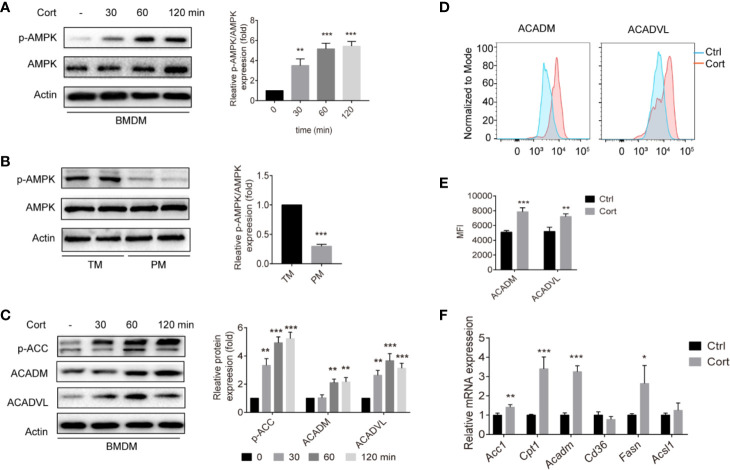
Corticosterone increases p-AMPK levels and FA metabolism in BMDM. **(A, C)** BMDM were stimulated with corticosterone (50 ng/ml) from 30–120 min, the levels of AMPK/p-AMPK **(A)**, and p-ACC, ACADM, and ACADVL **(C)** were measured by Western blot. In each case, representative blots and average relative expression levels (determined by band intensity using Image J and normalized relative to BMDM control sample) are shown. **(B)** TM and PM were isolated and pooled from two mice (two mice for one sample, two samples were detected), p-AMPK/AMPK expression was measured by Western blot. Representative images of three independent experiments are shown. Relative amounts of p-AMPK detected on the blots were quantified as described in *Methods*. **(D, E)** BMDM were treated with corticosterone (50 ng/ml) for 7 days, cells were fixed and labeled for ACADM and ACADVL before analysis by flow cytometry. Representative MFI histogram **(D)** and the mean MFI **(E)** is shown (n = 4 per group). **(F)** The mRNA levels of FA metabolism genes were quantified by qRT-PCR and normalized against *Gapdh*. The data represent the means ± SD. Results are representative of four to five independent experiments. Welch’s t-test, *p < 0.05, **p < 0.01, ***p < 0.001 as indicated.

Having observed that corticosterone treatment induces an M2 polarization of BMDM that is metabolically similar to TM, we next aimed to identify the molecular mechanisms involved. The AMPK signaling pathway plays an important role in polarizing macrophages to an anti-inflammatory M2 phenotype (29); therefore we measured levels of phosphorylated AMPK (p-AMPK) in untreated BMDM and compared them with BMDM treated with corticosterone for 30–120 min. We found that both the abundance of p-AMPK and the ratio of p-AMPK to AMPK significantly increased in a time-dependent manner following corticosterone treatment (**Figure 2A**). When we compared these AMPK levels to those in a population enriched in primary TM, again we saw a striking resemblance between corticosterone-treated BMDM and TM, which exhibited high absolute levels of p-AMPK and a high ratio of p-AMPK to AMPK; while in contrast PM exhibited significantly less p-AMPK and lower ratios between p-AMPK and AMPK (**Figure 2B**). This suggested that TM express high levels of p-AMPK constitutively, while PM do not, and that BMDM require corticosterone to activate AMPK.

Having shown that corticosterone treatment induces M2-like metabolic profiles in BMDM (**Figure 1D**) as well as inducing AMPK activation, we next asked how corticosterone impacted the AMPK-mediated fatty acid oxidation (FAO) pathway. As expected, corticosterone treatment of BMDM significantly increased the expression of key FAO enzymes including phosphorylated acetyl-CoA carboxylase (p-ACC), acyl-coenzyme A dehydrogenase (ACADM), and very long-chain specific acyl-CoA dehydrogenase (ACADVL) (**Figures 2C–E**). In addition, the mRNA levels of the FA transport gene transcript carnitine palmitoyltransferase 1 (*Cpt1*), the FA oxidation related gene transcripts acetyl-CoA carboxylase 1 (*Acc1*) and *Acadm*, and the FA synthesis gene transcript fatty acid synthase (*Fasn)* were also up-regulated after corticosterone stimulation (**Figure 2F**). Together, these data show that corticosterone treatment of BMDM induces AMPK activation and reprograms macrophages towards fatty acid metabolism, which is needed for M2 polarization. Moreover, similar relative levels of p-AMPK are present in corticosterone-treated BMDM and TM in the steady state.

### AMPK Inhibition Reduces M2 Phenotypic and Metabolic M2 Polarization in BMDM and TM

**Figure 3:**
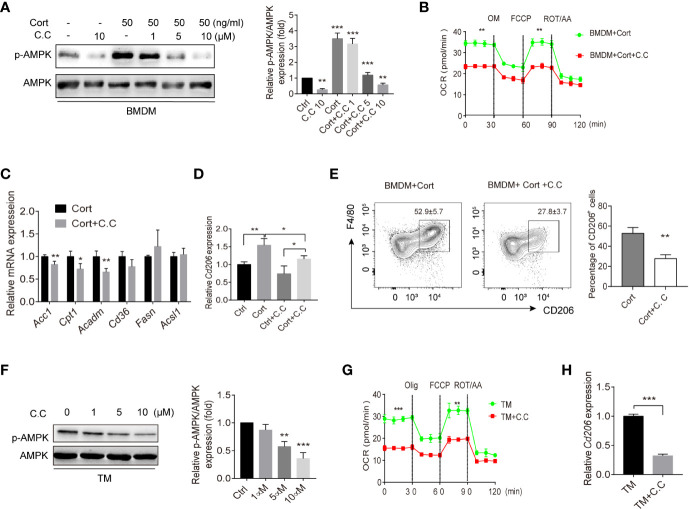
AMPK inhibition suppresses M2 polarization of BMDM and TM. **(A)** Corticosterone (50 ng/ml, 7 days)-polarized BMDM were incubated with the AMPK inhibitor Compound C (1–10μM, 3h); p-AMPK protein expression was detected by Western blot. Bar blots represent the relative p-AMPK/AMPK protein level, which was determined from the band intensity using ImageJ software, and normalized relative to the BMDM control samples. **(B)** The OCR of corticosterone-polarized BMDM (same samples as in **Figure 1D**) were compared with BMDM+Compound C treatment. **(C, D)** The relative mRNA levels of fatty acid metabolism genes and *Cd206* in BMDM and corticosterone stimulated (50 ng/ml, 3 h) Expression of *Acc1*, *Cpt1*, *Acadm*, cluster of differentiation 36 (*Cd36*), *Fasn*, acyl-coA synthetase long chain family member 1 (*Acsl1*) in BMDM were quantified by qRT-PCR and normalized against *Gapdh* (means ± SD Welch’s t-test for C, one way ANOVA for D.) **(E)** BMDM were stimulated with corticosterone for 7 days with/without daily Compound C (C. C; 10μM) administration. Levels of F4/80 and CD206 were identified by flow cytometry and the representative plot is shown. Bar blot shows the average of CD206^+^ cell percentage (means ± SD, Welch’s t-test). **(F)** TM were incubated with Compound C (1–10 μM, 3 h) and p-AMPK protein expression was detected by Western blot and quantified by using ImageJ **(G)**. The OCR of enriched populations of TM (same samples as in **Figure 1E**) and Compound C pre-treated TM (10 μM, 0.5 h) were measured in XF-96 assay medium and normalized against protein concentration **(H)**. The relative expression of *Cd206* in TM treated with or without Compound C was detected by qRT-PCR and normalized against *Gapdh*. In all graphs, the error bars represent SEM, whereas results designated with * were significant (P < 0.05), ** (P < 0.005) or (***p < 0.001). These experiments were repeated three times, Welch’s t-test was used.

To confirm the necessity of AMPK activation for the metabolic and phenotypic M2 polarization of corticosterone-treated BMDM, we conducted a series of experiments using the AMPK inhibitor Compound C. We first confirmed that Compound C treatment reversed corticosterone-mediated AMPK activation, while leaving overall AMPK levels unaffected, in BMDM (**Figure 3A**). We then asked what effect inhibiting AMPK activation would have on the observed metabolic polarization of BMDM. Compound C treatment significantly lowered OCR in corticosterone polarized BMDM (**Figure 3B**), and significantly inhibited the expression of the FAO-associated genes *Acc1*, *Cpt1*, and *Acadm* (**Figure 3C**). Moreover, Compound C treatment approximately halved the expression of the M2 macrophage marker CD206 at both the gene (**Figure 3D**) and population frequency (**Figure 3E**) level in corticosterone-treated BMDM. Collectively, these data showed that corticosterone-induced BMDM phenotypic and metabolic M2 polarization is AMPK dependent.

To understand whether AMPK activation was playing a parallel role in primary TM, we repeated the same analyses using these cells with PM for comparison. Again, we saw that Compound C treatment reduced p-AMPK levels in a dose-dependent fashion (**Figure 3F**). Similar to corticosterone-treated BMDM, we found that inhibition of AMPK activation led to significantly reduced OCR (**Figure 3G**), as well as significant reductions in *Cd206* expression (**Figure 3H**). Taken together, these results show that AMPK inhibition in BMDM and TM results in reduced FAO and decreased expression of the M2 marker *Cd206*. In BMDM this is accompanied by lowered expression of FAO-associated genes and a reduced frequency of cells showing M2 phenotypic polarization.

### AMPK Is Required for Anti-Inflammatory Cytokine Production in Corticosterone-Polarized BMDM and in TM

**Figure 4:**
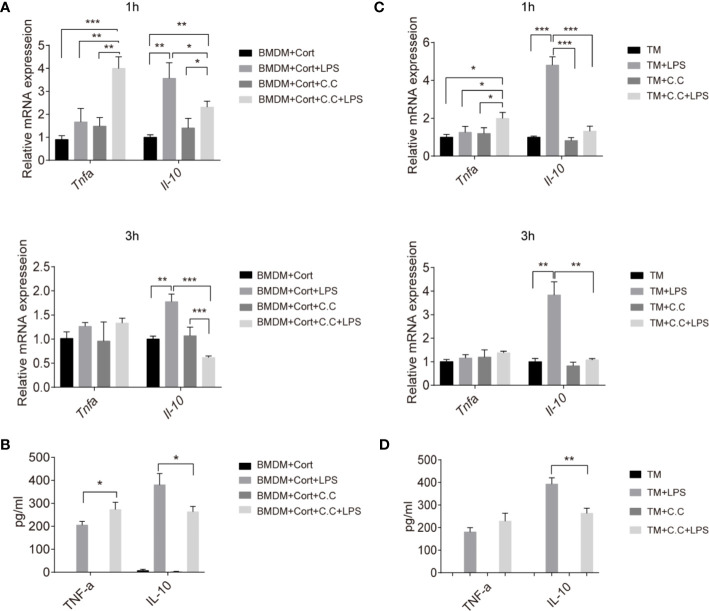
Inhibition of inflammatory cytokine production in M2 phenotype BMDM and TM by Compound C. Enriched populations of TM **(C, D)** and corticosterone-induced BMDM **(A, B)** were pre-treated with Compound C (C. C, 0.5 μM, 30 min), then stimulated with LPS (1 ug/ml, 1 and 3 h, respectively). **(A, C)** The effects of AMPK inactivation on expression of *Il10* and *Tnfa* were examined by qRT-PCR. **(B, D)** After pretreatment with Compound C and LPS (3 h) cell supernatants were collected and the protein levels of TNF-α and IL-10 were measured by ELISA. Results are representative of three to four independent experiments. *p < 0.05; **p < 0.01; *p <0.001 as indicated. One-way ANOVA.

Having shown that the phenotype and metabolic profile of corticosterone-polarized BMDM and TM relies on AMPK activation, we next investigated whether AMPK activation was needed for M2-associated cytokine production. Quantitative RT-PCR of cytokine gene expression showed that *Tnfa* transcript levels were elevated in corticosterone-treated BMDM in the presence or absence of the bacterial inflammatory stimulus LPS after 1 h, and were unaffected by Compound C treatment (**Figure 4A**). Expression levels equaled at 3 h post-LPS stimulation. In contrast, expression of the anti-inflammatory cytokine *Il10* gene was significantly increased following LPS treatment at 1 and 3 h, and inhibited by Compound C (**Figure 4A**). At the protein level, TNFα levels were high 3 h after LPS treatment of corticosterone-treated BMDM, and the addition of Compound C further enhanced this effect (**Figure 4B**). IL-10 protein levels confirmed the mRNA data, showing significant AMPK-activation-dependent induction following LPS exposure (**Figure 4B**). Thus, AMPK activation is crucial for the induction of immunosuppressive IL-10 in corticosterone-polarized BMDM in response to challenge with a common bacterial ligand.

We next asked whether there was evidence for comparable mechanisms in primary TM. Again, *Tnfa* expression was not induced by LPS exposure of these cells, while instead abundant *Il-10* transcription was stimulated (**Figure 4C**). This is consistent with previous studies and confirms the immunosuppressive features of TM (10). Compound C pre-treatment significantly reduced both the *Il10* transcript and protein level in enriched populations of TM upon LPS stimulation (**Figures 4C, D**), suggesting that AMPK is important for expression of this key immunosuppressive cytokine by TM in response to a bacterial inflammatory stimulus.

### Corticosterone Treatment Ameliorates UPEC-Elicited Orchitis *In Vivo*

**Figure 5:**
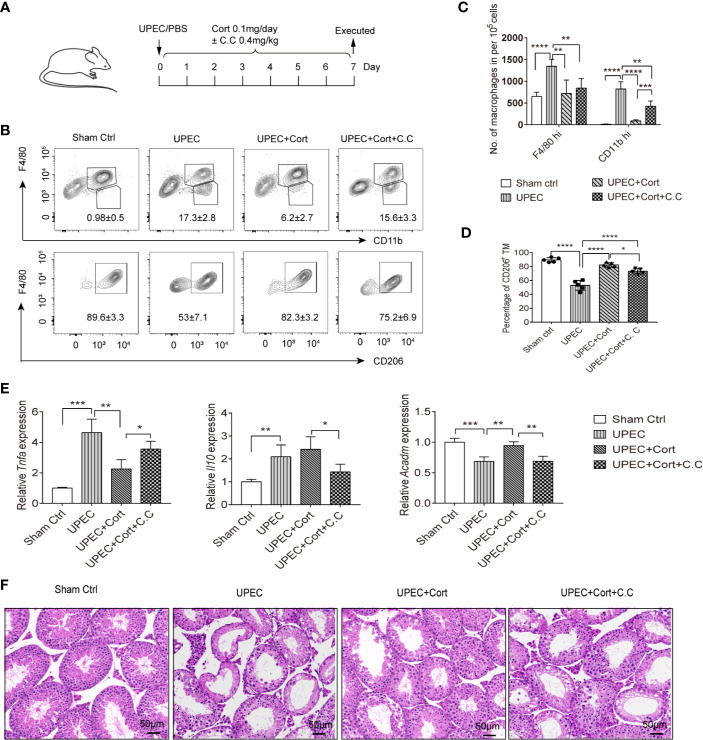
Corticosterone treatment protects testis from UPEC-induced orchitis. **(A)** WT C57BL/6J mice were injected with saline or uropathogenic *Escherichia coli* into the vas deferens. Mice were then injected daily with corticosterone (0.1mg/day) ± Compound C (C.C, 0.4 mg/kg). Seven days post-infection mice were sacrificed and macrophage populations were measured in cells from testes by flow cytometry. Data are representative of two independent experiments. **(B)** Mean percentages of F4/80^hi^CD11b^lo^ resident TM, F4/80^lo^CD11b^hi^ monocyte-derived macrophages (upper panel) and the F4/80^hi^ CD206^+^ M2 TM (lower panel) in testes of the different treatment groups are presented. **(C)** The number of resident F4/80^hi^ macrophages and CD11b^hi^ monocyte-derived macrophages in testes of the different treatment groups is presented. **(D)** Percentage of CD206-expressing TM among F4/80^hi^CD11b^lo^ resident macrophages in the different groups is shown; **(E)** TM were isolated and the relative expression of *Tnfa*, *Il10*, and *Acadm* were detected by RT-PCR. Mean ± SD; n = 5 for each group). The unpaired one-way ANOVA was employed for statistical analysis. *** p < 0.001, **p < 0.01, * p < 0.01. **(F)** Day 7 testicular tissue sections from each treatment group were stained with hematoxylin and eosin (magnification = 20× objective).

The data so far have confirmed that corticosterone induces BMDM to develop an M2 phenotype, metabolic state, and immunosuppressive cytokine response, dependent on AMPK activation. We next wanted to ask about the possible roles of corticosterone *in vivo*. Therefore, the effects of corticosterone treatment on the inflammatory immune response and resulting testicular tissue impairment was assessed in a murine model of uropathogenic *Escherichia coli* (UPEC)-elicited orchitis, besides the role of AMPK (**Figure 5A**). Seven days after UPEC injection into the vas deferens, mice were sacrificed, immune cell suspensions were analyzed by flow cytometry, and testicular sections taken for histology. We found that UPEC induced a marked increase in frequency (**Figure 5B**) and absolute number (**Figure 5C**) of both F4/80^hi^ resident TM and CD11b^hi^ monocyte-derived macrophages in testis, consistent with the inflammation caused by UPEC infection. However, in mice treated with corticosterone these increases were significantly less (**Figures 5B, C**), suggesting an anti-inflammatory function of corticosterone in the testis. By adding Compound C to the UPEC+corticosterone group, we uncovered a critical role for AMPK activation in mediating CD11b^hi^ macrophage recruitment into the UPEC-inflamed testis (**Figures 5B, C**). We then looked at M2 marker expression in the F4/80^+^ TM macrophages in each treatment group, and found that CD206 expression was significantly decreased by UPEC, but reinstated to control levels when corticosterone was applied (**Figures 5C, D**). AMPK activation was partially responsible for the change in frequency of CD206-expressing TM (**Figure 5D**). Moreover, compared to the UPEC treatment group, *Tnfa* expression was downregulated and *Acadm* expression was upregulated in enriched populations of TM isolated from the corticosterone treatment group—effects that were reversed by the addition of Compound C (**Figure 5E**). These data show that corticosterone reduced UPEC-induced inflammation in the murine testis by maintaining the immunosuppressive function of TM. When we examined control testis sections using H&E staining, we found a profound loss of germ cells in the seminiferous tubules (**Figure 5F**), consistent with the impaired spermatogenesis that often accompanies bacterial orchitis. In contrast, mice infected with UPEC and treated with corticosterone were less severely affected (**Figure 5F**). Again, the effects of corticosterone were partially reversed by Compound C treatment (**Figure 5F**), indicating an important role of AMPK signaling in corticosterone-mediated amelioration of UPEC-elicited orchitis. Taken together, corticosterone treatment protected testes from inflammation-associated tissue damage *via* a pathway involving AMPK activation and associated with maintaining TM M2 phenotype as well as reducing the abundance of infiltrating monocyte-derived macrophages.

## Discussion

The current study shows that corticosterone can help maintain the TM immunosuppressive phenotype under bacterial challenge, and that the AMPK pathway is responsible for part of the TM-mediated immunosuppression. In line with these findings, corticosterone treatment mediated profound changes to BMDM energy metabolism—inhibiting glycolysis and inducing FAO—to promote M2 polarization. Further experiments indicated that AMPK activation plays a major role in corticosterone-polarized M2 macrophage phenotype and function. Moreover, corticosterone suppresses the pro-inflammatory response of BMDM, instead promoting IL-10 secretion in response to LPS. Similarly, *in vivo* in a UPEC elicited-orchitis mouse model, corticosterone treatment prevented UPEC-induced decreases in M2 TM and reduced tissue impairment in a partially AMPK-dependent manner. These findings highlight an important role for AMPK *in vivo* in TM, and demonstrate the therapeutic potential for corticosterone in maintaining the anti-inflammatory properties of TM and the immune privilege of the testis.

In previous studies in rats it was observed that TM play a crucial role in regulation of testicular inflammation by presenting an anti-inflammatory phenotype and secreting high amounts of IL-10 (10, 11). We further identified corticosterone as a main contributor to maintaining TM’s immunosuppressive phenotype and function (10, 12). The anti-inflammatory function of corticosterone has been intensively investigated in many leucocytes, including macrophages, where it changes the phenotype and function of these cells (30). A recent study also showed that corticosterone attenuates LPS-induced inflammatory cytokine IL-1β secretion in BV2 mouse microglia-like cells (31), which occupy a similarly immune-privileged niche in the brain as TM do in the testis. However, until now the effects of corticosterone on murine TM and during testicular infection was unknown. It needs to be noted that isolated primary TM show a purity of approx. 80% and are thus cautiously referred to as a TM enriched population with likely contaminants consisting of germ cells and Leydig cells that attach tightly to TM.

In this study, corticosterone treatment polarized BMDM towards a F4/80^+^CD206^+^ M2 anti-inflammatory phenotype with reduced TNF-α secretion and increased IL-10 production *in vitro*, similar to TM, suggesting a crucial role of corticosterone in mediating macrophage M2 polarization.

The underlying mechanism of corticosterone-mediated BMDM M2 polarization remained elusive. Recent advances in the immune-metabolism field have shown that the metabolic pathways and phenotypic polarization of macrophages are closely intertwined: macrophages can switch their phenotype and function by changing their metabolic pathways in response to the tissue microenvironment or upon encountering inflammatory cues (32, 33). Energy metabolism is not only a source of energy but also provides signals for macrophage polarization (17). Our study confirmed that, typically, M2 TM as well as corticosterone-polarized M2 BMDM showed increased FAO and decreased glycolysis, compared with M1 macrophage PM. Of note, we found that corticosterone upregulated expression of several FA metabolism-related genes in BMDM by activating AMPK, the master regulator of metabolism. The AMPK inhibitor Compound C reversed the anti-inflammatory property of corticosterone-polarized BMDM by inhibiting FAO. These results further confirmed that a switch of metabolic pathway can regulate macrophage phenotype and function and shed new light on the understanding of corticosterone-induced macrophage M2 polarization. However, how AMPK-mediates FA metabolism still needs further investigation.

Accumulating evidence has shown that AMPK activation exhibits anti-inflammatory features mainly by activating SIRT1 and PGC-1a, and by inhibiting several inflammatory pathways including NF-kB and AP-1 (34, 35). In rheumatoid arthritis, SIRT1/adenosine monophosphate enhances anti-inflammatory M2 macrophage polarization by activating AMPK signaling (36). Similarly, in this study we observed that corticosterone-mediated activation of AMPK polarized BMDM towards an immunosuppressive phenotype *in vitro* and supported maintenance of the M2 TM population *in vivo* under bacterial challenge. However, the mechanism of corticosterone-mediated AMPK activation is still not fully understood. In alignment with our findings, it has been recently reported that another glucocorticoid, dexamethasone, can improve therapeutic outcomes in a mouse model of UPEC-mediated epididymitis by inhibiting the immune response and tissue damage (37). Moreover, our *in vivo* data further demonstrated that corticosterone treatment ameliorates UPEC-elicited orchitis by increasing the percentage of M2 macrophages in the TM population; while Compound C treatment reduced the percentage of M2 macrophages, suggesting that the anti-inflammatory function of corticosterone depends at least partially on AMPK activation. In addition, corticosterone treatment significantly reduced the inflammatory CD11b^hi^ monocytes population in the infected testis, and importantly, reduced inflammation-associated tissue damage.

Taken together, we have uncovered the underlying mechanisms of corticosterone-induced M2 BMDM polarization, in which AMPK activation-induced FAO plays a key role. We also evidence similar pathways in TM both *ex vivo* and *in vivo*, where corticosterone therapy ameliorated the tissue-damaging effects of UPEC-elicited orchitis by maintaining the population of M2 TM. Thus this work has uncovered key metabolic pathways and mediators underpinning the anti-inflammatory polarization of TM, and shed new light on the development of innovative therapeutics for orchitis patients.

## Data Availability Statement

The original contributions presented in the study are included in the article/**Supplementary Material**. Further inquiries can be directed to the corresponding authors.

## Ethics Statement

The animal study was reviewed and approved by Ethics Committee of ZhengZhou University.

## Author Contributions

Conceptualization: SB, AM, and MW. Methodology: MW, ZZ, ZJ, YuZ, YiZ and YY. Formal analysis: MW, YY, and ZJ. Supervision and fund acquisition: MW, ZZ, and ZQ. Writing—review and editing: ZZ and MW. All authors contributed to the article and approved the submitted version.

## Conflict of Interest

The authors declare that the research was conducted in the absence of any commercial or financial relationships that could be construed as a potential conflict of interest.

## Supplementary Material

Supplementary Figure 1 The purities of TM and PM were analyzed by flow cytometry. Isolated primary TM and PM were stained with anti-CD45, anti-F4/80, and anti-CD11b antibody, followed by FACS analysis. Representative plots are shown.
