# Supplementary figures and images for: Corticosterone Enhances the AMPK-Mediated Immunosuppressive Phenotype of Testicular Macrophages During Uropathogenic Escherichia coli Induced Orchitis
Source: Front Immunol. 2020 Dec 8;11:583276. doi: 10.3389/fimmu.2020.583276 (PMC7752858; doi:10.3389/fimmu.2020.583276)

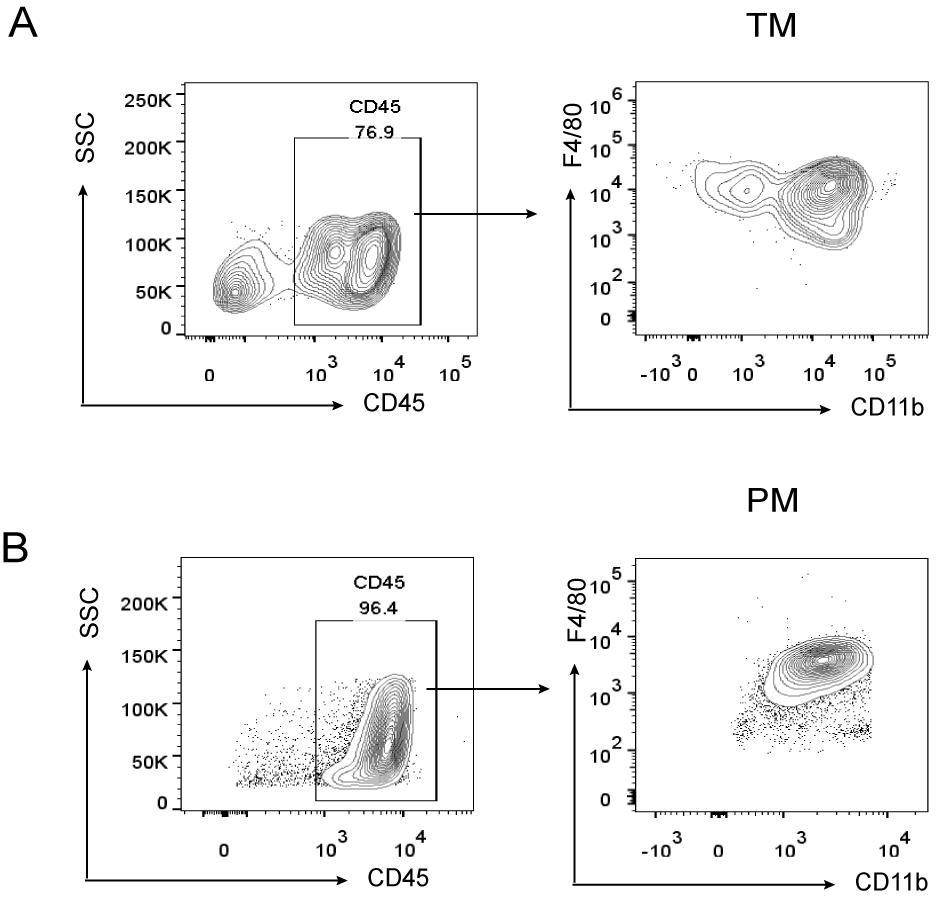

Supplement: Supplementary Figure 1 — The purities of TM and PM were analyzed by flow cytometry. Isolated primary TM and PM were stained with anti-CD45, anti-F4/80, and anti-CD11b antibody, followed by FACS analysis. Representative plots are shown. [file Image_1.tif]
